# Methylation of the Claudin 1 Promoter Is Associated with Loss of Expression in Estrogen Receptor Positive Breast Cancer

**DOI:** 10.1371/journal.pone.0068630

**Published:** 2013-07-03

**Authors:** Francescopaolo Di Cello, Leslie Cope, Huili Li, Jana Jeschke, Wei Wang, Stephen B. Baylin, Cynthia A. Zahnow

**Affiliations:** The Sidney Kimmel Comprehensive Cancer Center at Johns Hopkins, Johns Hopkins University School of Medicine, Baltimore, Maryland, United States of America; Institut national de la santé et de la recherche médicale, France

## Abstract

Downregulation of the tight junction protein claudin 1 is a frequent event in breast cancer and is associated with recurrence, metastasis, and reduced survival, suggesting a tumor suppressor role for this protein. Tumor suppressor genes are often epigenetically silenced in cancer. Downregulation of claudin 1 *via* DNA promoter methylation may thus be an important determinant in breast cancer development and progression. To investigate if silencing of claudin 1 has an epigenetic etiology in breast cancer we compared gene expression and methylation data from 217 breast cancer samples and 40 matched normal samples available through the Cancer Genome Atlas (TCGA). Moreover, we analyzed claudin 1 expression and methylation in 26 breast cancer cell lines. We found that methylation of the claudin 1 promoter CpG island is relatively frequent in estrogen receptor positive (ER+) breast cancer and is associated with low claudin 1 expression. In contrast, the claudin 1 promoter was not methylated in most of the ER-breast cancers samples and some of these tumors overexpress claudin 1. In addition, we observed that the demethylating agents, azacitidine and decitabine can upregulate claudin 1 expression in breast cancer cell lines that have a methylated claudin 1 promoter. Taken together, our results indicate that DNA promoter methylation is causally associated with downregulation of claudin 1 in a subgroup of breast cancer that includes mostly ER+ tumors, and suggest that epigenetic therapy to restore claudin 1 expression might represent a viable therapeutic strategy in this subtype of breast cancer.

## Introduction

Tight junctions are responsible for some of the defining characteristics of epithelial cells and tissues. They form a tight seal between adjacent cells that restricts paracellular transport (gate function) and separates the apical and basolateral domains of the plasma membrane to maintain cell polarity (fence function). These dynamic structures respond to a variety of environmental, physiological and pharmacological cues, and it is well accepted that their dysfunction or disruption, and the ensuing loss of tissue organization contribute to the development and progression of cancer [1–3]. The three principal components of tight junctions are occludin, claudins and junctional adhesion molecule (JAM), but only claudins are considered indispensable for tight junction formation [1–5]. Twenty-seven human claudins have thus far been identified [6]. The specific pattern of expression depends on tissue type and stage of development [1,2,6], or on the specific disease associated with dysregulation and tight junction disruption [1–3,7]. Several studies have analyzed the role of claudin 1 (CLDN1) in epithelial physiology as well as in cancer development and metastasis (reviewed by Gupta and Ryan [1] and Myal, Leygue and Blanchard [2]). Both downregulation and overexpression of claudin 1 have been associated with tumorigenesis, suggesting that claudin 1 may alternatively function as a tumor suppressor or as an oncogene. For example, in papillary thyroid tumors, oral squamous cell carcinoma, melanoma, ovarian, colon and gastric cancer overexpression of claudin 1 has been associated with aggressiveness and increased malignant phenotype. Conversely, in esophageal, prostate and lung cancer loss of claudin 1 correlates with cancer progression, invasion, metastasis, and shorter disease-free survival [2]. In breast cancer, expression of claudin 1 appears to vary according to the molecular subtype [2,7,8]. Claudin 1 overexpression has been observed in some estrogen receptor negative (ER-), basal-like breast cancers [7,8]. However, the most prominent role for claudin 1 in breast cancer appears to be that of tumor suppressor. Claudin 1 expression is low or absent in most breast cancer samples and cell lines, in sharp contrast with the normal mammary epithelium where this protein has a typical apicolateral membrane localization [2,7–11]. Moreover, loss of claudin 1 correlates with breast cancer recurrence, metastasis, and reduced survival [12,13]. Claudin 1 downregulation seems more prominent in ER+ [8,14] or ER+/HER2+ luminal [7,14] breast cancers, but is also a feature of some basal-like breast cancers including those belonging to the “claudin-low” subtype, which have low levels of claudin 1, 3, 4, 7 and 8 and poor prognosis [7,15]. Additional evidence of a claudin 1 tumor suppressor role comes from *in vitro* studies showing that anti-claudin 1 antibodies promote transformation of MCF‑12A breast epithelial cells [16] and re-expression of claudin 1 induces apoptosis in tridimensional cultures of MDA‑MB‑361 breast cancer cells [17]. The mechanisms responsible for decreased claudin 1 expression are not completely understood and neither the coding sequence nor the promoter region of claudin 1 appear to be mutated in either sporadic or hereditary breast cancer patients, or in breast cancer cell lines [10]. Methylation of CpG dinucleotides within a gene promoter region is a well characterized epigenetic mechanism responsible for the silencing of tumor suppressor genes [18]. DNA promoter methylation has been associated with the silencing of claudin 4 in bladder carcinoma [19] and claudin 6 and 7 in breast cancer [20,21], and methylation of the claudin 1 promoter has been reported in the colon cancer cell line HCT116 [22], which does not express claudin 1. Moreover, elevated methylation and low expression of claudin 1 were observed in breast cancer samples from The Cancer Genome Atlas (TCGA) [23]. To investigate if claudin 1 downregulation has an epigenetic etiology in human breast cancer we compared gene expression and methylation data from 217 patient samples available through TCGA [23] and from 26 breast cancer cell lines analyzed in our laboratory. Our analysis shows that DNA promoter methylation is associated with downregulation of claudin 1 in a subgroup of breast cancers that include mostly ER+ tumors. Moreover we demonstrated the causality of this link by showing that treating human breast cancer cell lines with the DNA demethylating drugs azacitidine and decitabine results in increased claudin 1 expression and in its localization to the cell surface, which can potentially lead to the restoration of normal polarized growth [4,5] or to tumor suppression *via* apoptosis [17].

## Materials and Methods

### Cell lines

All cells lines were obtained from ATCC (Manassas, VA, USA) except SUM 149PT and SUM 159PT [24] (generously donated by Dr. Steve Ethier), and EFM19 and EFM192A (DSMZ collection, Braunschweig, Germany; generously donated by Dr. Dennis Slamon). Standard culture conditions are summarized in Supplementary Table S1; three-dimensional (3D) matrigel cultures were prepared as previously described [25].

### Analysis of gene expression and methylation

Gene expression and DNA methylation data obtained from human primary breast cancer samples and matched normal breast tissue samples using the Agilent G4502A (Agilent Technologies) and the Infinium HumanMethylation450 (Illumina, Inc.) microarray platforms was downloaded from TCGA [23]. Gene expression and DNA methylation analysis of breast cancer cell lines was performed at the microarray core of The Sidney Kimmel Comprehensive Cancer Center at Johns Hopkins using the Agilent G4112F and the Infinium HumanMethylation450 microarrays as previously described [26]. The microarray data was analyzed using R (http://www.r-project.org) and the limma package in Bioconductor [27] as previously described [26] and deposited in the GEO database under accession numbers GSE44836 and GSE44837. Statistical tests and generation of heat maps, scatterplots, and boxplots, were performed in R using standard functions included with the base distribution. Quantitative Real-Time PCR analysis (qRT-PCR) of claudin 1 mRNA expression in breast cancer cell lines was performed on a 7500 Real-Time PCR System, using cDNA generated with the High Capacity cDNA Reverse Transcription Kit, TaqMan Gene Expression Master Mix, the Hs00221623_m1 TaqMan Gene Expression Assays and the Human RPLP0 Endogenous Control (Life Technologies, Grand Island, NY, USA). Methylation specific PCR (MSP) was performed on bisulfite-treated DNA (EZ DNA methylation kit, Zymo Research, Irvine, CA, USA) as previously described [28]; primers were designed using MSPPrimer [29] and the sequences are given in Supplementary Table S2.

### Flow cytometry

Fluorescence-activated cell sorting (FACS) was performed in duplicate on a BD Bioscience FACSCalibur. Single cells suspensions obtained by enzymatic digestion using 0.05% trypsin (Life Technologies, Grand Island, NY, USA) were labeled with a monoclonal antibody for human claudin 1 conjugated with Alexa Fluor 488 (Clone 421203; R&D Systems, Minneapolis, MN, USA) according to the manufacturer’s instructions.

## Results and Discussion

**Figure 1:**
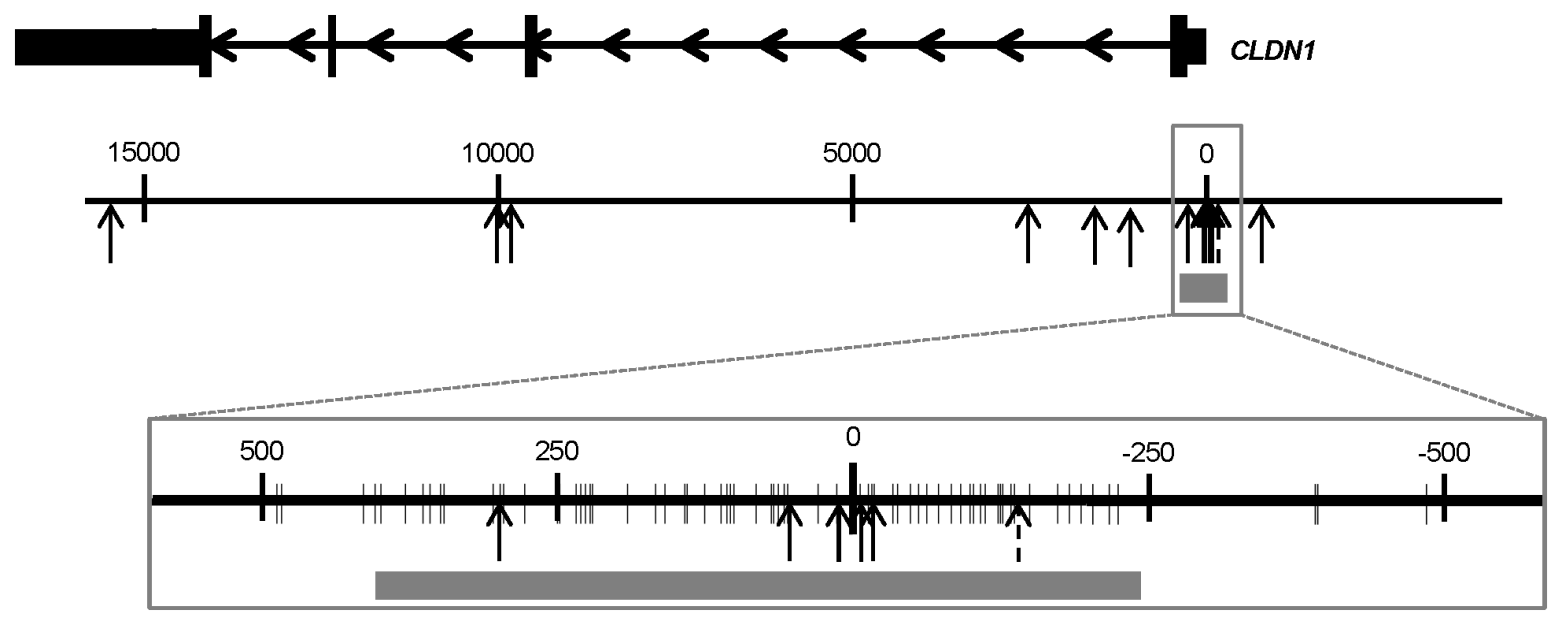
Map of the claudin 1 CpG island and associated methylation probes. Claudin 1 (*CLDN1*) mRNA is depicted on top with arrows to indicate the orientation of the gene; the thicker sections represent the coding sequence, the medium sections represent the untranslated exon regions, and the thinner sections represent the introns. The genomic region encompassing claudin 1 is represented in two different scales; the numbers indicate the distance from the TSS in nucleotides. The vertical arrows mark the position of the probes included in the Infinium HumanMethylation450 microarray. The probe excluded from the analysis because of a SNP is indicated as a dashed arrow. The grey box indicates the location of the CpG island. Individual CpG sites are marked as thin vertical marks in the magnified map.

Gene expression data from the Agilent G4502A microarray with matched DNA methylation data from Infinium HumanMethylation450 microarray was available for 217 breast cancer patient samples in TCGA [23]. Matched normal tissue was available for 40 of these samples. The HumanMethylation450 microarray includes 13 probes for claudin 1. Six of these probes are located within a CpG island that extends across the transcription start site (TSS), one of the remaining seven probes is upstream of the CpG island and six are downstream (Figure 1). One probe within the CpG island (cg24550865) was excluded from the analysis because of a SNP in the target sequence.

**Figure 2:**
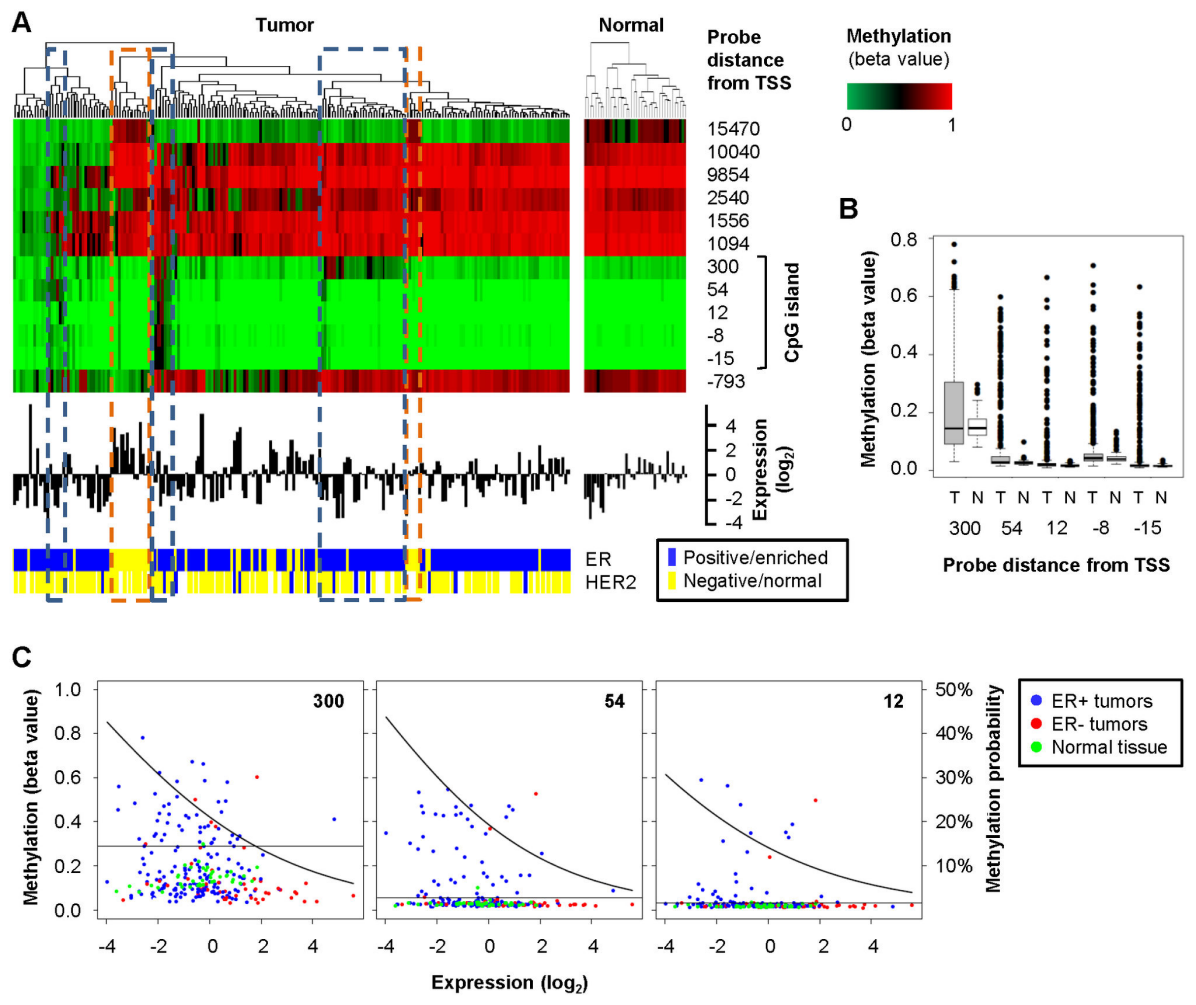
Methylation of claudin 1 is associated with loss of expression in human breast cancer. A: cluster analysis and heat map of claudin 1 methylation (top), histogram of claudin 1 mRNA expression (middle), and ER/HER2 status (bottom) for 217 samples of invasive breast carcinoma and 40 matched normal tissue samples from TCGA; the turquoise dashed boxes mark three clusters of samples with methylation of the claudin 1 CpG island; the orange dashed boxes mark two clusters of ER-, basal-like samples. B: box plot of methylation of the claudin 1 CpG island in the same TCGA samples; T, tumors; N, normal. C: dot plots of claudin 1 gene expression and methylation for the three CpG island sites that have significant Spearman’s correlation coefficients (the position of each probe is noted in the upper right corner); the horizontal line marks the mean methylation value of normal samples plus three standard deviations, which is taken as threshold for methylation; the curve represents the probability of methylation as a function of expression level estimated by fitting a logistic regression model using the logit link function. DNA methylation is shown as beta values, which are the ratio of methylated DNA to total DNA: a value of 1 indicates 100% methylation.

In many of the breast cancer samples and in all the normal tissue samples claudin 1 methylation is low within the CpG island and high outside the CpG island, which is not indicative of epigenetic silencing (Figure 2A). Claudin 1 expression varies considerably in these samples and might be controlled by transcription factors such as Slug and Snail, which are key markers of epithelial-mesenchymal transition (EMT) and have been shown to affect claudin 1 [30]. Most ER-breast cancer samples have low claudin 1 methylation at the promoter CpG island and some of them have the highest claudin 1 expression in the whole cohort, consistent with previous reports of elevated claudin 1 in ER-, basal-like breast cancer [2,7,8]. These ER-, basal-like clusters also stand out for having stronger DNA methylation than other breast cancer samples at the most distal site, 15470 nt from the transcription start of claudin 1. However, methylation at this particular site is present in some of the normal breast tissue samples as well (Figure 2A). Interestingly, we also observed clusters of breast cancer samples with methylation of the claudin 1 promoter CpG island (Figure 2A). These included mostly ER+ samples with lower claudin 1 expression, which is indicative of epigenetic silencing. Since we have not analyzed normal tissue from cancer-free individuals we cannot exclude that the DNA methylation pattern of the matched normal breast tissue is also somewhat altered because of a cancer field effect [31,32]. However, methylation of the claudin 1 CpG island is generally low in all the normal breast tissue samples with very few outliers, which is indicative of a normal methylation pattern for an expressed gene. In contrast, the breast cancer samples are much more variable and include samples with significantly higher methylation at each CpG island site (Figure 2B, P < 0.00001 by Student’s t-test). To estimate the proportion of breast cancer samples with methylation of the claudin 1 CpG island we considered methylated any sample with a beta value (ratio of methylated DNA to total DNA) exceeding the corresponding mean value for normal breast tissue by more than three standard deviations. Based on this threshold, which was established individually for each probe, 38.5% of the breast cancer samples have at least one methylated site within the claudin 1 CpG island and 17.4% are methylated at two or more sites. There was significant inverse correlation between gene methylation and expression for three of the five sites, indicating that increased methylation at these sites is associated with reduced claudin 1 expression (Table 1). To evaluate the importance of DNA methylation as a mechanism of silencing claudin 1 expression in breast cancer, we used a logistic regression model estimating the probability of methylation as a function of the gene expression for these three sites (Figure 2C). Depending on the specific site, DNA methylation accounts for 30-40% of the tumors with the lowest level of gene expression, decreasing to approximately 20% at median expression and to about 5% for tumors with the highest expression levels (Figure 2C). These data indicate that in the absence of CpG island methylation claudin 1 is regulated by other factors, but it is also downregulated or silenced through the methylation of its CpG island in a subset of breast cancers.

**Table 1:** Methylation of the claudin 1 promoter CpG island in invasive breast carcinomas and normal breast tissue.

**Probe ID**	**Distance from TSS**	**Methylation (beta value)**		**Methylated tumors^*b*^**	**Correlation with expression^*c*^**	
		**Normal^*a*^**	**Tumor^*a*^**			
cg15105660	300	0.15 ± 0.05	0.20 ± 0.15	26%	-0.19 *P*=0.00395	
cg08770122	54	0.03 ±0.01	0.07 ± 0.11	21%	-0.24 *P* =0.00037	
cg14310674	12	0.02 ± 0.00	0.04 ± 0.08	13%	-0.19 *P* =0.00407	
cg21919136	-8	0.05 ± 0.02	0.07 ± 0.10	12%	-0.11 *P* =0.09656	
cg07661818	-15	0.02 ± 0.00	0.04 ± 0.09	14%	-0.13 *P* =0.06255	

^a^ Average ± standard deviation;

^b^ beta values exceeding the mean value of normal breast tissue by more than three standard deviations;

^a^ Spearman’s rank correlation coefficient and Student's t-test. Beta values are the ratio of methylated DNA to total DNA: a value of 1 indicates 100% methylation.

**Figure 3:**
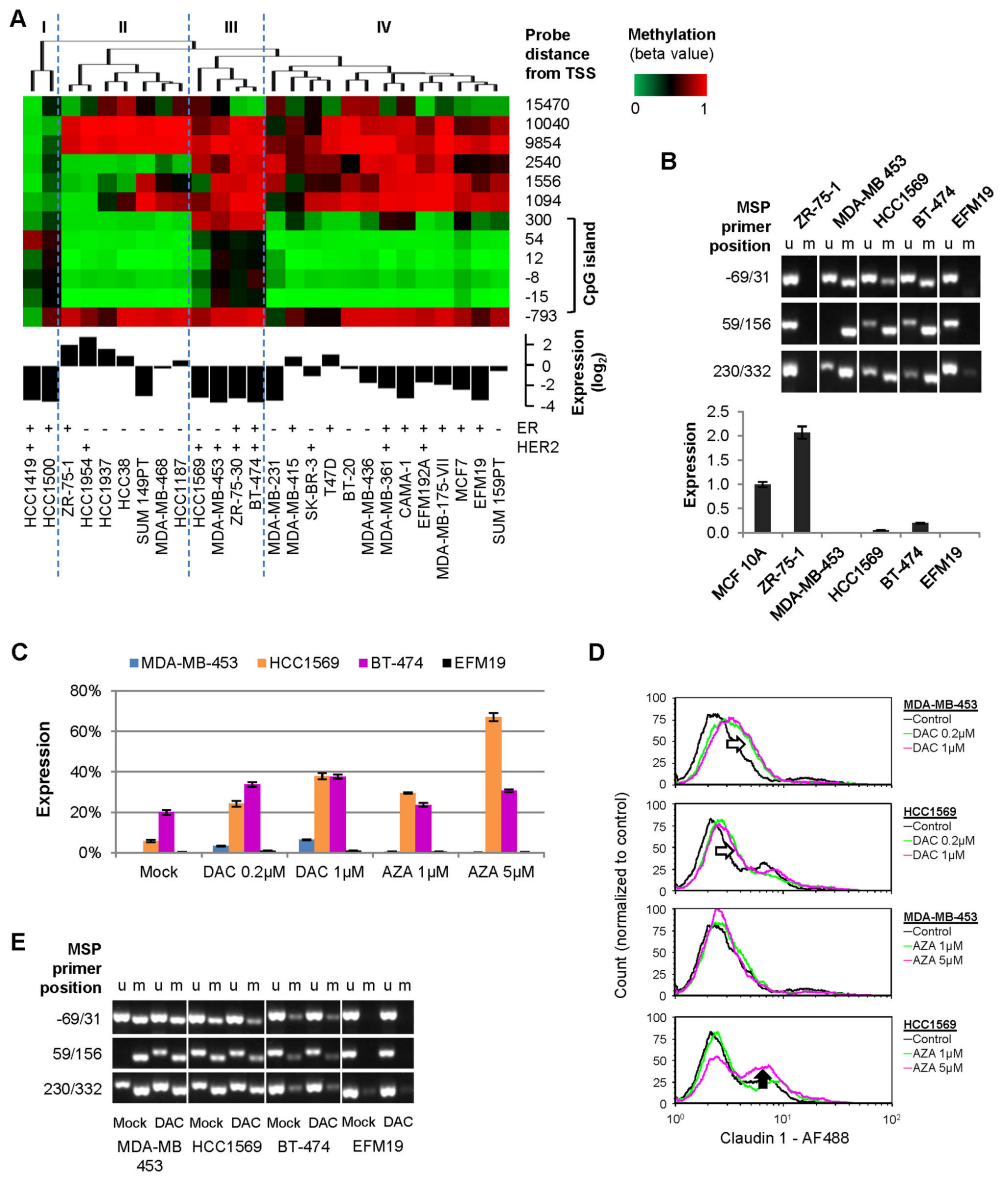
Methylation of claudin 1 is associated with loss of expression in breast cancer cell lines. A: cluster analysis and heat map of claudin 1 methylation (top), histogram of claudin 1 mRNA expression (middle), and ER/HER2 status [39] (bottom) in breast cancer cell lines; specific clusters are marked with roman numbers and separated by dashed lines; gene expression is relative to the average of all cell lines. B: Validation of claudin 1 promoter methylation by MSP (top), and gene expression by qRT-PCR (bottom) in selected cell lines; the non-tumorigenic mammary epithelial cell line MCF 10A was added as reference for gene expression; u, unmethylated; m, methylated. C: qRT-PCR analysis of claudin 1 expression in breast cancer cell lines treated with decitabine (DAC) or azacitidine (AZA); claudin 1 expression in MCF 10A cells was used as reference (100% expression). D: FACS analysis of claudin 1 surface expression in breast cancer cell lines treated with decitabine (DAC) or azacitidine (AZA); the empty arrows indicate increase fluorescence (claudin 1 positivity) in treated cells, the filled arrow indicates an increase in the proportion of a defined cell population that expresses claudin 1. E: MSP analysis of claudin 1 promoter methylation in breast cancer cell lines treated with 1µM DAC; u, unmethylated; m, methylated.

To obtain experimental evidence for the causal role of DNA methylation in claudin 1 silencing we replicated our analysis in breast cancer cell lines, which can be treated *in vitro* with demethylating agents to achieve re-expression [18]. We analyzed twenty-six breast cancer cell lines using the Agilent G4112F gene expression microarray and the Infinium HumanMethylation450 microarray (Figure 3A). Similar to what we observed in the breast cancer samples, methylation of the CpG island correlates with claudin 1 expression in the cell lines, with the most significant Spearman’s rank coefficient at the sites located 300 and 54 nt after the transcription start (ρ = -0.6656, and -0.7361 respectively, *P*<0.001). Overall, the methylation pattern of these breast cancer cell lines resembles that of the TCGA cohort. Half of them form a cluster characterized by low methylation in the CpG island and high methylation outside the CpG island (Figure 3A, group IV). Claudin 1 expression varies considerably in this group, which includes cell lines of various ER/HER2 status. The second larger cluster is composed mostly of ER-, basal-like cell lines, which feature some loss of methylation at sites downstream of the CpG island (Figure 3A, group II). This group includes cell lines with the highest expression of claudin 1, which may be representative of the claudin-high subtype of breast cancer [2,7]. Two cell lines (HCC1419 and HCC1500) have very low claudin 1 expression and are under-methylated at the distal sites and partially methylated at the CpG island (Figure 3A, group I). The methylation patterns of these two cell lines somewhat resemble that of some of the TCGA breast cancer samples (Figure 2A, leftmost samples), although TCGA samples that are demethylated at non-CpG island sites are mostly devoid of methylation within the CpG island as well. Four cell lines stand out for being methylated at the claudin 1 CpG island: HCC1569, MDA‑MB‑453, ZR‑75‑30, and BT‑474 (Figure 3A, group III). Although two of these cell lines are ER-, this methylation pattern resembles that of the clusters of claudin 1-low, mostly ER+ breast cancer samples from TCGA, in which claudin 1 appear to be epigenetically silenced. Consistently, the microarray data indicate that expression of claudin 1 is very low in these four cell lines. The relative abundance of ER-cell lines in this group might be a result of the high proportion of ER-cell lines in the cohort compared to the frequency of human ER-breast cancer. All the cell lines of this group are HER2+; however we did not identify a similar correlation with HER2 status in the TCGA samples. We validated claudin 1 promoter methylation by MSP, and gene expression by qRT-PCR for three of these cell lines plus lines ZR‑75‑1 and EFM19 from group II and IV, respectively (Figure 3B). The non-tumorigenic mammary epithelial cell line MCF 10A was added as reference for claudin 1 expression. The analysis confirmed that both ZR‑75‑1 and EFM19 are not methylated at the claudin 1 promoter CpG island, and while ZR‑75‑1 cells express twice as much claudin 1 mRNA as MCF 10A, the expression of this gene in EFM19 is almost undetectable (Figure 3B). Consistent with the microarray data, claudin 1 expression is low or absent in MDA‑MB‑453, HCC1569 and BT-474 cells, which are methylated to different degrees at the claudin 1 CpG island (Figure 3B).

We used the demethylating agents decitabine (DAC) and azacitidine (AZA) to further demonstrate that DNA methylation is responsible for claudin 1 silencing in the breast cancer cell lines MDA‑MB‑453, HCC1569 and BT‑474. Decitabine and azacitidine are distinct cytidine analogs that are used clinically for the treatment of patients with myelodysplastic syndromes and acute myeloid leukemia (AML) [33,34]. As a ribonucleoside, azacitidine is incorporated into both RNA and DNA, while decitabine, a deoxyribonucleoside, is incorporated solely into DNA) [35]. Both act to inhibit DNA methyltransferases [36,37], and to induce demethylation of DNA and re-expression of methylated and silenced genes [38]; however, decitabine shows equivalent activity at doses approximately 2-10 fold lower than azacitidine [18]. Cultured cells were treated daily for three days with decitabine (0.2 µM and 1µM) or azacitidine (1µM and 5µM). Decitabine caused re-expression of claudin 1 in MDA‑MB‑453, HCC1569 and BT‑474 cells; however, HCC1569 and BT‑474 cells achieved the higher level of expression, corresponding to approximately 40% of that of MCF 10A cells (Figure 3C). This represents a 2-fold increase for BT‑474 cells and a 6.6-fold increase for HCC1569 (*P*<0.0001). MDA‑MB‑453 cells had the lowest basal expression of claudin 1 among these cell lines, and although the gene was upregulated over 400 times by treatment with 1µM decitabine (*P*<0.0001), the overall level of expression remained lower than in the other two cell lines (Figure 3C). Treatment with azacitidine resulted in a similar upregulation of claudin 1 and was most effective in HCC1569 cells where it caused an 11.6-fold increase in expression (*P*<0.0001). Similarly to decitabine, azacitidine upregulated claudin 1 in MDA‑MB‑453 cells (up to 38.7-fold, *P*<0.0001) but the overall amount of mRNA remained low compared to the other cell lines. Re-expression of claudin 1 protein on the surface of treated HCC1569 and MDA‑MB‑453 cells was confirmed by flow cytometry. Similar to what was observed by qRT-PCR analysis, decitabine caused an increase in claudin 1 signal in both cell lines (Figure 3D, empty arrows) and 5 µM azacitidine was again the most effective treatment in HCC1569 as it caused an increase in the proportion of claudin 1-positive cells compared to the untreated controls (Figure 3D, filled arrow). To confirm that the re-expression of claudin 1 could be ascribed to demethylation of its promoter CpG island, we analyzed claudin 1 methylation by MSP in the cells treated with 1µM decitabine, as this particular treatment had consistently caused re-expression in all the cell lines tested. MSP analysis shows that the MDA‑MB‑453 has the strongest methylation of the claudin 1 promoter among these cell lines, and although decitabine was effective, there was considerable residual methylation after treatment (Figure 3E). Conversely, in the less methylated HCC1569 and BT‑474 cells treatment results in a less pronounced upregulation but in a higher overall expression of claudin 1. Thus, the methylation status of the claudin 1 promoter correlates well with both basal expression and response to treatment, which is consistent with epigenetic regulation. As a control, we treated the EMF19 cell line, which is not methylated at the claudin 1 CpG island according to both the microarray and MSP analysis. As expected, neither drug had significant effect on claudin 1 expression in EMF19 cells (Figure 3C, E).

To investigate if treatment with decitabine or azacitidine affects the phenotype of these cells in a manner consistent with re-expression of claudin 1, we cultured HCC1569 and MDA‑MB‑453 cells in a 3D matrigel matrix. Untreated cells formed large undifferentiated cell aggregates characteristic of a transformed phenotype. In contrast, cells treated with either decitabine or azacitidine formed few if any 3D colonies (not shown). Although this assay cannot discriminate between the numerous anti-tumorigenic effects described for decitabine and azacitidine [26] and that specifically due to re-expression of claudin1, this result is consistent with the previous observation that re-expression of claudin 1 induces apoptosis in 3D breast cancer cell cultures [17].

## Conclusions

Our analysis of the TCGA data shows that there is a significant correlation between downregulation of claudin 1 expression and methylation of its promoter CpG island in a subset of breast cancer. In the absence of promoter methylation, the expression of claudin 1 varies considerably, and might be regulated by transcription factors such as Slug and Snail, as previously described [30]. Claudin 1 expression is highest in some ER-samples, which is consistent with previous reports showing elevated claudin 1 expression in this subtype of breast cancer [2,7,8]. In the samples that have a methylated claudin 1 promoter, claudin 1 expression is low or absent, which is consistent with epigenetic silencing. These are mostly ER+ samples, suggesting that claudin 1 promoter methylation is associated with this subtype in particular. Breast cancer cell lines recapitulate these phenotypes and we identified cell lines in which claudin 1 is silenced through promoter methylation as well as others in which claudin 1 is not silenced and is instead overexpressed. Treatment of claudin 1-negative cell lines with demethylating agents such as decitabine or azacitidine causes re-expression of claudin 1 mRNA as well as increased claudin 1 protein on the cell surface. Moreover, we observed that both decitabine and azacitidine disrupt the growth of breast cancer cells in matrigel. These drugs are known to have various anti-tumorigenic effects [26], thus we cannot conclude that this inhibition of growth is due specifically to tumor suppression by claudin 1. However, our results concordantly indicate that DNA promoter methylation is causally associated with downregulation of claudin 1 in a group of breast cancers that include mostly ER+ tumors. Given the supposed tumor suppressor role of claudin 1 in this subtype of breast cancer [7,8,14], epigenetic therapy to restore claudin 1 expression might represent a viable therapeutic strategy.

## Supporting Information

Table S1 Cell culture conditions. Cell lines were incubated in the indicated media at 37.0°C in 95% air, 5% carbon dioxide humid atmosphere.
(PDF)

Table S2 MSP primers. We thank Alexander Koch and Dr. Hariharan Easwaran for their assistance in the preliminary analysis of the TCGA data.
(PDF)

## References

[1] Gupta IR, Ryan AK (2010) Claudins: Unlocking the code to tight junction function during embryogenesis and in disease. Clin Genet 77: 314-325. doi:10.1111/j.1399-0004.2010.01397.x. PubMed: 20447145. 20447145 10.1111/j.1399-0004.2010.01397.x

[2] Myal Y, Leygue E, Blanchard AA (2010) Claudin 1 in breast tumorigenesis: Revelation of a possible novel "claudin high" subset of breast cancers. J Biomed Biotechnol: 2010: 956897 10.1155/2010/956897 PMC2871677 20490282

[3] Turksen K, Troy TC (2011) Junctions gone bad: Claudins and loss of the barrier in cancer. Biochim Biophys Acta 1816: 73-79. PubMed: 21515339. 21515339 10.1016/j.bbcan.2011.04.001

[4] Saitou M, Fujimoto K, Doi Y, Itoh M, Fujimoto T et al. (1998) Occludin-deficient embryonic stem cells can differentiate into polarized epithelial cells bearing tight junctions. J Cell Biol 141: 397-408. doi:10.1083/jcb.141.2.397. PubMed: 9548718. 9548718 10.1083/jcb.141.2.397 PMC2148457

[5] Furuse M, Sasaki H, Fujimoto K, Tsukita S (1998) A single gene product, claudin-1 or -2, reconstitutes tight junction strands and recruits occludin in fibroblasts. J Cell Biol 143: 391-401. doi:10.1083/jcb.143.2.391. PubMed: 9786950. 9786950 10.1083/jcb.143.2.391 PMC2132845

[6] Mineta K, Yamamoto Y, Yamazaki Y, Tanaka H, Tada Y et al. (2011) Predicted expansion of the claudin multigene family. FEBS Lett 585: 606-612. doi:10.1016/j.febslet.2011.01.028. PubMed: 21276448. 21276448 10.1016/j.febslet.2011.01.028

[7] Lu S, Singh K, Mangray S, Tavares R, Noble L et al. (2013) Claudin expression in high-grade invasive ductal carcinoma of the breast: Correlation with the molecular subtype. Mod Pathol 26: 485-495. doi:10.1038/modpathol.2012.187. PubMed: 23222490. 23222490 10.1038/modpathol.2012.187 PMC4000969

[8] Blanchard AA, Skliris GP, Watson PH, Murphy LC, Penner C et al. (2009). Claudins 1: 3 and 4 protein expression in ER negative breast cancer correlates with markers of the basal phenotype. Virchows Arch 454: 647-656 10.1007/s00428-009-0770-6 19387682

[9] Tokés AM, Kulka J, Paku S, Szik A, Páska C et al. (2005) Claudin-1, -3 and -4 proteins and mRNA expression in benign and malignant breast lesions: A research study. Breast Cancer Res 7: R296-R305. doi:10.1186/bcr983. PubMed: 15743508. 15743508 10.1186/bcr983 PMC1064136

[10] Krämer F, White K, Kubbies M, Swisshelm K, Weber BH (2000) Genomic organization of claudin-1 and its assessment in hereditary and sporadic breast cancer. Hum Genet 107: 249-256. doi:10.1007/s004390000375. PubMed: 11071387. 11071387 10.1007/s004390000375

[11] Gerhard R, Ricardo S, Albergaria A, Gomes M, Silva AR et al. (2012) Immunohistochemical features of claudin-low intrinsic subtype in metaplastic breast carcinomas. Breast 21: 354-360. doi:10.1016/j.breast.2012.03.001. PubMed: 22464177. 22464177 10.1016/j.breast.2012.03.001

[12] Morohashi S, Kusumi T, Sato F, Odagiri H, Chiba H et al. (2007) Decreased expression of claudin-1 correlates with recurrence status in breast cancer. Int J Mol Med 20: 139-143. PubMed: 17611630. 17611630

[13] Szasz AM, Tokes AM, Micsinai M, Krenacs T, Jakab C et al. (2011) Prognostic significance of claudin expression changes in breast cancer with regional lymph node metastasis. Clin Exp Metastasis 28: 55-63. doi:10.1007/s10585-010-9357-5. PubMed: 20963473. 20963473 10.1007/s10585-010-9357-5

[14] Ricardo S, Gerhard R, Cameselle-Teijeiro JF, Schmitt F, Paredes J (2012) Claudin expression in breast cancer: High or low, what to expect? Histol Histopathol 27: 1283-1295. PubMed: 22936447. 22936447 10.14670/HH-27.1283

[15] Herschkowitz JI, Simin K, Weigman VJ, Mikaelian I, Usary J et al. (2007) Identification of conserved gene expression features between murine mammary carcinoma models and human breast tumors. Genome Biol 8: R76. doi:10.1186/gb-2007-8-5-r76. PubMed: 17493263. 17493263 10.1186/gb-2007-8-5-r76 PMC1929138

[16] Kulawiec M, Safina A, Desouki MM, Still I, Matsui S et al. (2008) Tumorigenic transformation of human breast epithelial cells induced by mitochondrial DNA depletion. Cancer Biol Ther 7: 1732-1743. doi:10.4161/cbt.7.11.6729. PubMed: 19151587. 19151587 10.4161/cbt.7.11.6729 PMC2783327

[17] Hoevel T, Macek R, Swisshelm K, Kubbies M (2004) Reexpression of the TJ protein CLDN1 induces apoptosis in breast tumor spheroids. Int J Cancer 108: 374-383. doi:10.1002/ijc.11571. PubMed: 14648703. 14648703 10.1002/ijc.11571

[18] Jones PA, Baylin SB (2002) The fundamental role of epigenetic events in cancer. Nat Rev Genet 3: 415-428. PubMed: 12042769. 12042769 10.1038/nrg816

[19] Boireau S, Buchert M, Samuel MS, Pannequin J, Ryan JL et al. (2007) DNA-methylation-dependent alterations of claudin-4 expression in human bladder carcinoma. Carcinogenesis 28: 246-258. PubMed: 16829686. 16829686 10.1093/carcin/bgl120

[20] Osanai M, Murata M, Chiba H, Kojima T, Sawada N (2007) Epigenetic silencing of claudin-6 promotes anchorage-independent growth of breast carcinoma cells. Cancer Sci 98: 1557-1562. doi:10.1111/j.1349-7006.2007.00569.x. PubMed: 17645772. 17645772 10.1111/j.1349-7006.2007.00569.x PMC11158132

[21] Kominsky SL, Argani P, Korz D, Evron E, Raman V et al. (2003) Loss of the tight junction protein claudin-7 correlates with histological grade in both ductal carcinoma in situ and invasive ductal carcinoma of the breast. Oncogene 22: 2021-2033. doi:10.1038/sj.onc.1206199. PubMed: 12673207. 12673207 10.1038/sj.onc.1206199

[22] Ogoshi K, Hashimoto S, Nakatani Y, Qu W, Oshima K et al. (2011) Genome-wide profiling of DNA methylation in human cancer cells. Genomics 98: 280-287. doi:10.1016/j.ygeno.2011.07.003. PubMed: 21821115. 21821115 10.1016/j.ygeno.2011.07.003

[23] Cancer Genome Atlas Network (2012) Comprehensive molecular portraits of human breast tumours. Nature 490: 61-70

[24] Ignatoski KM, Ethier SP (1999) Constitutive activation of pp125fak in newly isolated human breast cancer cell lines. Breast Cancer Res Treat 54: 173-182. doi:10.1023/A:1006135331912. PubMed: 10424408. 10424408 10.1023/a:1006135331912

[25] Debnath J, Muthuswamy SK, Brugge JS (2003) Morphogenesis and oncogenesis of MCF-10A mammary epithelial acini grown in three-dimensional basement membrane cultures. Methods 30: 256-268. doi:10.1016/S1046-2023(03)00032-X. PubMed: 12798140. 12798140 10.1016/s1046-2023(03)00032-x

[26] Tsai HC, Li H, Van Neste L, Cai Y, Robert C et al. (2012) Transient low doses of DNA-demethylating agents exert durable antitumor effects on hematological and epithelial tumor cells. Cancer Cell 21: 430-446. doi:10.1016/j.ccr.2011.12.029. PubMed: 22439938. 22439938 10.1016/j.ccr.2011.12.029 PMC3312044

[27] Smyth GK, Speed T (2003) Normalization of cDNA microarray data. Methods 31: 265-273. doi:10.1016/S1046-2023(03)00155-5. PubMed: 14597310. 14597310 10.1016/s1046-2023(03)00155-5

[28] Herman JG, Graff JR, Myöhänen S, Nelkin BD, Baylin SB (1996) Methylation-specific PCR: A novel PCR assay for methylation status of CpG islands. Proc Natl Acad Sci U S A 93: 9821-9826. doi:10.1073/pnas.93.18.9821. PubMed: 8790415. 8790415 10.1073/pnas.93.18.9821 PMC38513

[29] Brandes JC, Carraway H, Herman JG (2007) Optimal primer design using the novel primer design program: MSPprimer provides accurate methylation analysis of the ATM promoter. Oncogene 26: 6229-6237. doi:10.1038/sj.onc.1210433. PubMed: 17384671. 17384671 10.1038/sj.onc.1210433

[30] Martínez-Estrada OM, Cullerés A, Soriano FX, Peinado H, Bolós V et al. (2006) The transcription factors slug and snail act as repressors of claudin-1 expression in epithelial cells. Biochem J 394: 449-457. doi:10.1042/BJ20050591. PubMed: 16232121. 16232121 10.1042/BJ20050591 PMC1408675

[31] Lewis CM, Cler LR, Bu DW, Zöchbauer-Müller S, Milchgrub S et al. (2005) Promoter hypermethylation in benign breast epithelium in relation to predicted breast cancer risk. Clin Cancer Res 11: 166-172. PubMed: 15671542. 15671542

[32] Yan PS, Venkataramu C, Ibrahim A, Liu JC, Shen RZ et al. (2006) Mapping geographic zones of cancer risk with epigenetic biomarkers in normal breast tissue. Clin Cancer Res 12: 6626-6636. doi:10.1158/1078-0432.CCR-06-0467. PubMed: 17121881. 17121881 10.1158/1078-0432.CCR-06-0467

[33] Cataldo VD, Cortes J, Quintás-Cardama A (2009) Azacitidine for the treatment of myelodysplastic syndrome. Expert Rev Anticancer Ther 9: 875-884. doi:10.1586/era.09.61. PubMed: 19589026. 19589026 10.1586/era.09.61

[34] Saba HI (2007) Decitabine in the treatment of myelodysplastic syndromes. Ther Clin Risk Manag 3: 807-817. PubMed: 18473005. 18473005 PMC2376088

[35] Yoo CB, Jones PA (2006) Epigenetic therapy of cancer: Past, present and future. Nat Rev Drug Discov 5: 37-50. doi:10.1038/nrd1930. PubMed: 16485345. 16485345 10.1038/nrd1930

[36] Stresemann C, Bokelmann I, Mahlknecht U, Lyko F (2008) Azacytidine causes complex DNA methylation responses in myeloid leukemia. Mol Cancer Ther 7: 2998-3005. doi:10.1158/1535-7163.MCT-08-0411. PubMed: 18790780. 18790780 10.1158/1535-7163.MCT-08-0411

[37] Ghoshal K, Datta J, Majumder S, Bai S, Kutay H et al. (2005) 5-aza-deoxycytidine induces selective degradation of DNA methyltransferase 1 by a proteasomal pathway that requires the KEN box, bromo-adjacent homology domain, and nuclear localization signal. Mol Cell Biol 25: 4727-4741. doi:10.1128/MCB.25.11.4727-4741.2005. PubMed: 15899874. 15899874 10.1128/MCB.25.11.4727-4741.2005 PMC1140649

[38] Stresemann C, Brueckner B, Musch T, Stopper H, Lyko F (2006) Functional diversity of DNA methyltransferase inhibitors in human cancer cell lines. Cancer Res 66: 2794-2800. doi:10.1158/0008-5472.CAN-05-2821. PubMed: 16510601. 16510601 10.1158/0008-5472.CAN-05-2821

[39] Finn RS, Dering J, Conklin D, Kalous O, Cohen DJ et al. (2009) PD 0332991, a selective cyclin D kinase 4/6 inhibitor, preferentially inhibits proliferation of luminal estrogen receptor-positive human breast cancer cell lines in vitro. Breast Cancer Res 11: R77. doi:10.1186/bcr2419. PubMed: 19874578. 19874578 10.1186/bcr2419 PMC2790859

