# Supplementary material for: Methylation of the Claudin 1 Promoter Is Associated with Loss of Expression in Estrogen Receptor Positive Breast Cancer
Source: PLoS One. 2013 Jul 3;8(7):e68630. doi: 10.1371/journal.pone.0068630 (PMC3701071; doi:10.1371/journal.pone.0068630)
Supplement: Table S1 — Cell lines were incubated in the indicated media at 37.0°C in 95% air, 5% carbon dioxide humid atmosphere. (PDF) [file pone.0068630.s001.pdf]

Table S1. Cell culture conditions. Cell lines were incubated in the indicated media at 37.0°C in 95% air, 5%carbon dioxide humid atmosphere.

Cell line	Base Medium	FBS	Additives
BT-20	MEM (Cellgro® 10-010)	10%	
BT-474	Hybri-Care Medium (ATCC® 46-X)	10%	1.5 g/L sodium bicarbonate
CAMA-1	MEM (Cellgro® 10-010)	10%	non-essential amino acids, 1mM sodium pyruvate
EFM19	RPMI-1640 (Cellgro® 10-040)	10%	
EFM192A	RPMI-1640 (Cellgro® 10-040)	10%	
HCC1187	RPMI-1640 (Cellgro® 10-040)	10%	
HCC1419	RPMI-1640 (Cellgro® 10-040)	10%	
HCC1500	RPMI-1640 (Cellgro® 10-040)	10%	
HCC1569	RPMI-1640 (Cellgro® 10-040)	10%	
HCC1937	RPMI-1640 (Cellgro® 10-040)	10%	
HCC1954	RPMI-1640 (Cellgro® 10-040)	10%	
HCC38	RPMI-1640 (Cellgro® 10-040)	10%	
MCF 10A	DMEM:F12 (Gibco® 11330)		20 ng/ml Human EGF, 100 ng/ml cholera toxin, 0.01 mg/ml bovine insulin, 500 ng/ml hydrocortisone, 5% horse serum
MCF7	MEM (Cellgro® 10-010)	10%	0.01 mg/ml bovine insulin
MDA-MB-175-VII	DMEM (Cellgro® 10-013)	10%	
MDA-MB-231	DMEM (Cellgro® 10-013)	10%	
MDA-MB-361	DMEM (Cellgro® 10-013)	20%	
MDA-MB-415	DMEM (Cellgro® 10-013)	15%	0.01 mg/ml bovine insulin, 10 ug/ml glutathione
MDA-MB-436	DMEM (Cellgro® 10-013)	10%	0.01 mg/ml bovine insulin, 16 ug/ml glutathione
MDA-MB-453	DMEM (Cellgro® 10-013)	10%	
MDA-MB-468	DMEM (Cellgro® 10-013)	10%	
SK-BR-3	McCoy's 5A, Mod. (Cellgro® 10-050)	10%	
SUM 149PT	DMEM:F12 (Gibco® 11330)	5%	0.005 mg/ml bovine insulin, 1 ug/ml hydrocortisone, 10mM HEPES
SUM 159PT	DMEM:F12 (Gibco® 11330)	5%	0.005 mg/ml bovine insulin, 1 ug/ml hydrocortisone, 10mM HEPES
T47D	RPMI-1640 (Cellgro® 10-040)	10%	0.01 mg/ml bovine insulin
ZR-75-1	RPMI-1640 (Cellgro® 10-040)	10%	
ZR-75-30	RPMI-1640 (Cellgro® 10-040)	10%	
