# Supplementary material for: Methylation of the Claudin 1 Promoter Is Associated with Loss of Expression in Estrogen Receptor Positive Breast Cancer
Source: PLoS One. 2013 Jul 3;8(7):e68630. doi: 10.1371/journal.pone.0068630 (PMC3701071; doi:10.1371/journal.pone.0068630)
Supplement: Table S2 — We thank Alexander Koch and Dr. Hariharan Easwaran for their assistance in the preliminary analysis of the TCGA data. (PDF) [file pone.0068630.s002.pdf]

Table S2. MSP primers.

CLDN1(1)_US	5‘-GTTAGGGGGTGTTTTTTGGTTGTTG-3‘
CLDN1(1)_UAS	5‘-ACTACAATTACTCCCAAACTCAAAAACTAAAACA-3‘
CLDN1(1)_MS	5‘-GGGGCGTTTTTCGGTTGTC-3‘
CLDN1(1)_MAS	5‘-TACGATTACTCCCAAACTCGAAAACTAAAACG-3‘
CLDN1(2)_US	5‘-TTTTAGTGTTGTTTTGGGTGTGGATTTTAATTTTG-3‘
CLDN1(2)_UAS	5‘-AAAATAACTAAACCCCACAAAAAAAATTAAAACA-3‘
CLDN1(2)_MS	5‘-TTTCGGGCGCGGATTTTAATTTC-3‘
CLDN1(2)_MAS	5‘-AAATAACTAAACCCCGCGAAAAAAATTAAAACG-3‘
CLDN1(3)_US	5‘-GGGTGTTTGAGTGAGTTATGGTTAATGTG-3‘
CLDN1(3)_UAS	5‘-CCTCCACTAAAACAAAACAATACTAACAATAACA-3‘
CLDN1(3)_MS	5‘-TCGAGCGAGTTATGGTTAACGC-3‘
CLDN1(3)_MAS	5‘-CTCCACTAAAACAAAACAATACTAACGATAACG-3‘
